# The Influence of COVID-19 on Out-Hospital Cardiac Arrest Survival Outcomes: An Updated Systematic Review and Meta-Analysis

**DOI:** 10.3390/jcm10235573

**Published:** 2021-11-27

**Authors:** Karol Bielski, Agnieszka Szarpak, Miłosz Jaroslaw Jaguszewski, Tomasz Kopiec, Jacek Smereka, Aleksandra Gasecka, Przemysław Wolak, Grazyna Nowak-Starz, Jaroslaw Chmielewski, Zubaid Rafique, Frank William Peacock, Lukasz Szarpak

**Affiliations:** 1 Research Unit, Polonia University, 4/6 Pulaskiego Str., 42-200 Czestochowa, Poland; karol.bielski@meditrans.waw.pl; 2 Provincial Emergency Medical Service Dispatcher, 22 Poznanska, 00-685 Warsaw, Poland; 3 Institute of Outcomes Research, Maria Sklodowska-Curie Medical Academy, 12 Solidarnosci Av., 03-411 Warsaw, Poland; agnieszkaszarpak2019@gmail.com; 4 1st Department of Cardiology, Medical University of Gdansk, 3A Sklodowskiej-Curie Str., 80-210 Gdansk, Poland; jamilosz@gmail.com; 5 First Chair and Department of Cardiology, Medical University of Warsaw, 1A Banacha Str., 02-097 Warsaw, Poland; t.j.kopiec@gmail.com (T.K.); gaseckaa@gmail.com (A.G.); 6 Department of Emergency Medical Service, Wroclaw Medical University, 6 Bartla Str., 52-443 Wroclaw, Poland; Jacek.Smereka@umed.wroc.pl; 7 Research Unit, Polish Society of Disaster Medicine, P.O. Box 78, Raszyn, 05-090 Warsaw, Poland; 8 Institute of Medicine, Jan Kochanowski University of Kielce, 5 Zeromskiego Str., 25-369 Kielce, Poland; przemyslaw.wolak@ujk.edu.pl; 9 Institute of Health Sciences, Jan Kochanowski University of Kielce, 5 Zeromskiego Str., 25-369 Kielce, Poland; gnowakstarz@ujk.edu.pl; 10 College of Rehabilitation, 49 Kasprzaka Str., 01-234 Warsaw, Poland; jaroslaw.chmielewski@ios.home.pl; 11 Henry JN Taub Department of Emergency Medicine, Baylor College of Medicine Houston, Ben Taub Hospital, 1504 Taub Loop, Houston, TX 77030, USA; zubaidrafique@gmail.com (Z.R.); frankpeacock@gmail.com (F.W.P.); 12 Research Unit, Maria Sklodowska-Curie Bialystok Oncology Center, 12 Ogrodowa Str., 15-027 Bialystok, Poland

**Keywords:** SARS-CoV-2, cardiopulmonary resuscitation, coronavirus disease 2019, out-of-hospital cardiac arrest, outcome, pandemic

## Abstract

Cardiopulmonary resuscitation in patients with out-of-hospital cardiac arrest (OHCA) is associated with poor prognosis. Because the COVID-19 pandemic may have impacted mortality and morbidity, both on an individual level and the health care system as a whole, our purpose was to determine rates of OHCA survival since the onset of the SARS-CoV2 pandemic. We conducted a systematic review and meta-analysis to evaluate the influence of COVID-19 on OHCA survival outcomes according to the PRISMA guidelines. We searched the literature using PubMed, Scopus, Web of Science and Cochrane Central Register for Controlled Trials databases from inception to September 2021 and identified 1775 potentially relevant studies, of which thirty-one articles totaling 88,188 patients were included in this meta-analysis. Prehospital return of spontaneous circulation (ROSC) in pre-COVID-19 and COVID-19 periods was 12.3% vs. 8.9%, respectively (OR = 1.40; 95%CI: 1.06–1.87; *p* < 0.001). Survival to hospital discharge in pre- vs. intra-COVID-19 periods was 11.5% vs. 8.2% (OR = 1.57; 95%CI: 1.37–1.79; *p* < 0.001). A similar dependency was observed in the case of survival to hospital discharge with the Cerebral Performance Category (CPC) 1–2 (6.7% vs. 4.0%; OR = 1.71; 95%CI: 1.35–2.15; *p* < 0.001), as well as in the 30-day survival rate (9.2% vs. 6.4%; OR = 1.63; 95%CI: 1.13–2.36; *p* = 0.009). In conclusion, prognosis of OHCA is usually poor and even worse during COVID-19.

## 1. Introduction

The COVID-19 pandemic has negatively impacted health systems around the world and adversely affected cardiopulmonary resuscitation of out-of-hospital cardiac arrests (OHCA) [1,2]. As of 27 October 2021, more than 244 million cases of COVID-19 have been identified worldwide, with the number of deaths exceeding 4.9 million. Although most patients with COVID-19 recover without extensive intervention, a minority require intensive cardiac and respiratory support, ranging from oxygen therapy to extracorporeal membrane oxygenation [3].

The risk of exposure to SARS-CoV-2 infection to medical personnel, as well as those administering first aid during cardiac arrest, poses a challenge [4,5,6]. Until the widespread introduction of a vaccination program for medical personnel, this risk was of paramount concern. Although the risk is lower after the recent availability of effective COVID-19 vaccines, the lack of complete postvaccination protection and the emergence of new variants have made it necessary to alter protocols and provide protective equipment to medical personnel on an ongoing basis [7]. Many institutions and scientific societies have thus modified resuscitation protocols where the avoidance of invasive ventilation is preferred to protect medical staff [8]. Others have proposed an early termination of resuscitation in patients with COVID-19 [9].

These changes may have contributed to the recently increased number of out-of-hospital sudden cardiac arrest cases [3]. The reason for the increased number of cases is not only due to severe respiratory failure but also cardiac and vascular complications during and after infection [5]. Ultimately, cardiopulmonary resuscitation in patients with OHCA is associated with poor prognosis, and the COVID-19 pandemic may have worsened morbidity and mortality by impacting individual health and by crowding the healthcare system [9,10,11,12]. Critically ill COVID-19 patients may experience cardiac arrest, not only during acute hospitalization but also during rehabilitation and postrecovery periods [13].

Our previously published meta-analysis [14] suggests that suspicion or diagnosis of COVID-19 at OHCA is associated with a lower rate of shockable rhythms and a reduced survival to hospital discharge (SHD) rate. As there has been a tremendous amount of data reported since our prior publication, we performed an updated systematic review of the literature and meta-analysis to evaluate the influence of COVID-19 on OHCA survival outcomes.

## 2. Materials and Methods

This manuscript was prepared following the recommendations of the Preferred Reported Items for Systematic Reviews and Meta-Analysis (PRISMA) guidelines [15] (Table S3). A protocol of this meta-analysis has not been registered.

### 2.1. Search Strategy

Before commencing the study, all reviewers agreed on the analysis methods and the inclusion and exclusion criteria to be used. Two reviewers (K.B. and M.P.) independently performed a comprehensive literature search using PubMed, Scopus, Web of Science and Cochrane Central Register for Controlled Trials databases. The most recent search was performed on 10 October 2021. The search was conducted using the terms: “COVID-19” OR “SARS-CoV-2” AND “heart arrest” OR “cardiac arrest” OR “circulation arrest” OR “out-hospital cardiac arrest” OR “OHCA” OR “CA” OR “resuscitation” OR “CPR” OR “return of spontaneous circulation” OR “ROSC” OR “pulseless electrical activity” OR “asysto *” OR “pulseless ventric * tachycardia” OR “heart ventric * fibrillation” OR “cardiac ventric * fibrillation”. Additionally, a manual search of references listed in retrieved articles and reviews was also performed. All references were saved in an EndNote (End Note, Inc., Philadelphia, PA, USA) library used to identify the duplicates.

### 2.2. Eligibility Criteria

Studies included in this meta-analysis met the following PICOS criteria: (1) Participants: patients >18 years of age with out-of-hospital cardiac arrest due to any cause; (2) Intervention: cardiac arrest in COVID-19 period: (3) Comparison: cardiac arrest in pre-COVID-19 period; (4) Outcomes: detailed information for survival; (5) Study Design: randomized controlled trials, quasirandomized or observational studies comparing cardiac arrest during and before the COVID-19 period for their effects in patients with cardiac arrest. Finally, we excluded papers not containing comparator groups, conference or poster papers, reviews, case reports or articles not containing original data.

### 2.3. Data Extraction

Two reviewers (K.B. and J.C.) independently extracted data from each article that met the inclusion criteria. Disagreements among the authors regarding values or analysis assignments were resolved through discussion with the third reviewer (L.S.). Reviewers were careful to avoid the inclusion of data from duplicate publications. The data extracted from each study included the (1) study characteristics (i.e., first author’s name, year of publication, study location, study design, inclusion and exclusion criteria, primary findings); (2) participant characteristics in each group (i.e., number of participants, age, sex, comorbidities); (3) survival outcomes (i.e., the return of spontaneous circulation, survival to hospital admission with spontaneous circulation (SHA), survival to hospital discharge or survival to hospital discharge with good neurological outcome defined as 1 or 2 grade in Cerebral Performance Categories Scale).

### 2.4. Outcomes

We evaluated the following outcomes in our analysis, based on consensus among the content experts in our group, regarding important outcomes. The primary outcome was survival to hospital discharge (SHD), or 30 days, whichever came first. Secondary outcomes were the return of spontaneous circulation (ROSC), recurrence of cardiac arrest, survival with favorable neurologic status (defined as survival with Cerebral Performance Category (CPC) 1 or 2) and CPR parameters (i.e., bystander witnessed, bystander CPR).

### 2.5. Quality Assessment

A systematic assessment of bias in the included studies was performed using the Cochrane criteria [16,17]. For this purpose, a tool for Risk of Bias in Non-randomized Studies-of Interventions (ROBINS-I) [18] was used. ROBINS-I examines 7 domains of bias due to: (1) confounding; (2) selection of participants; (3) the classification of interventions; (4) deviations from intended interventions; (5) missing data; (6) measurement of outcomes; and (7) the selection of the reported result. The overall ROBINS-I judgment at the domain and study levels was attributed according to the criteria specified in the ROBVIS tool [19]. The risk of bias (RoB) was performed independently by two reviewers (T.K. and K.B.); disagreements were resolved by a third reviewer (L.S.) if necessary.

### 2.6. Statistical Analysis

The Mantel–Haenszel method was used to analyze dichotomous outcomes, and results are reported as odds ratios (ORs) or risk ratios (RRs) with a 95% confidence interval (CI). Continuous outcome differences were analyzed using an inverse variance model with a 95% CI, and values are reported as mean difference (MD). When the continuous outcome was reported in a study as median, range and interquartile range, we estimated means and standard deviations using the formula described by Hozo et al. [16].

We quantified heterogeneity in each analysis by the tau-squared and I-squared statistics. Heterogeneity was detected with the chi-squared test with *n*–1 degree of freedom, which was expressed as I^2^. Values of I^2^ > 50% and >75% were considered to indicate moderate and significant heterogeneity among studies, respectively. A random-effects model was used to pool study results independently of the *p*-value for heterogeneity or I^2^ [20]. All *p*-values were two-tailed and considered significant if *p* < 0.05. Statistical analysis was performed using Review Manager (ver. 5.4, Nordic Cochrane Centre, The Cochrane Collaboration, Copenhagen, Denmark). To evaluate the potential for publication bias, we plotted values against associated standard errors [21] and used Begg’s test to assess the symmetry of the resulting funnel plot [22]. We considered publication bias to be present at *p* < 0.1 in the asymmetry test. However, when a limited number of studies (<10) were included in the analysis, publication bias was not evaluated.

## 3. Results

### 3.1. Characteristics of Studies Included in the Meta-Analysis

**Figure 1:**
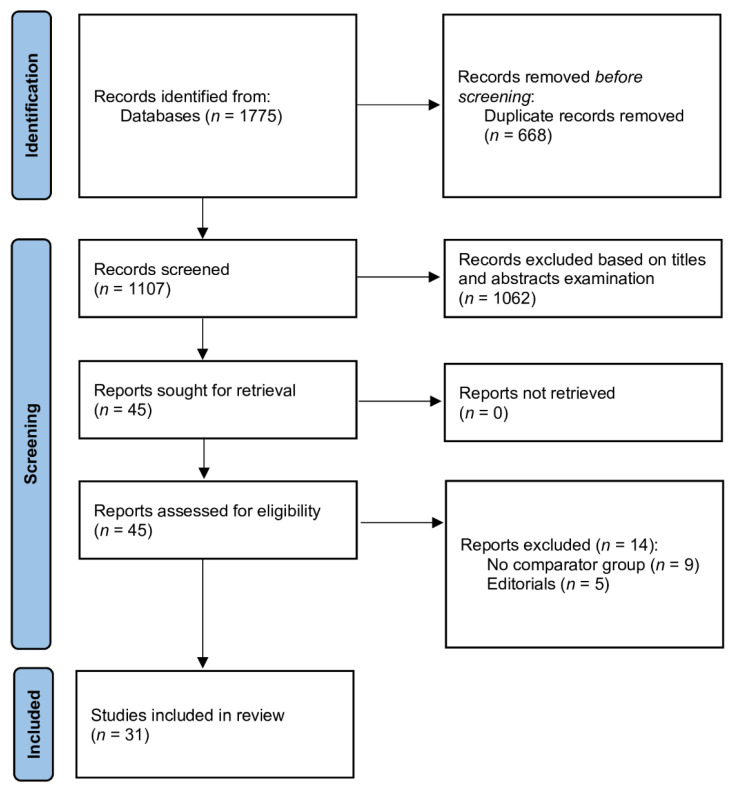
Database search and selection of studies according to PRISMA guidelines.

We searched 1775 potentially relevant studies in the databases (PubMed, Scopus, Web of Science and Cochrane Central). After excluding 668 duplicates, 1107 studies were screened using titles and abstracts; of those, 1062 studies were excluded. After full-text assessment for eligibility, 14 were excluded because they did not meet the inclusion criteria. Finally, thirty-one articles, including 88,188 patients, were included in this meta-analysis [23,24,25,26,27,28,29,30,31,32,33,34,35,36,37,38,39,40,41,42,43,44,45,46,47,48,49,50,51,52,53]. A flow diagram showing stages of database search and study selection is shown in Figure 1.

Twenty-seven studies reported OHCA outcomes in pre- vs. intra-COVID-19 periods [23,24,26,27,28,29,31,33,35,37,38,39,40,41,42,44,45,46,47,48,49,50,51,52,53], and OHCA outcomes in SARS-CoV-2-positive vs. -negative patients were reported in seven trials [25,30,32,34,36,43,51]. Characteristics of the individual studies are presented in Tables S1 and S2. The mean age of patients suffering from OHCA in the pre-COVID-19 period was 66.0 ± 17.3 years compared to 67.8 ± 17.1 years for patients since the COVID-19 pandemic.

The Cochrane risk of bias of the included studies is shown in Figures S1 and S2. The overall risk of bias was judged as low in twenty-nine studies [23,24,25,26,27,28,29,30,31,32,34,35,36,37,38,39,40,41,42,43,44,45,46,48,49,50,51,52,53], and reviewers indicate some concerns on the other two [33,47].

### 3.2. Resuscitation Characteristics in Pre- vs. Intra-COVID-19 Periods

**Figure 2:**
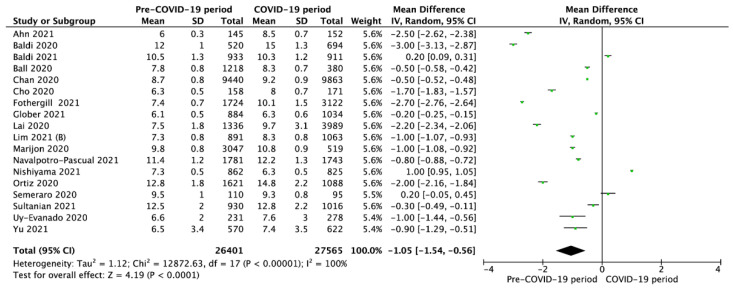
Forest plot of time to Emergency Medical Service arrival in pre- vs. intra-COVID-19 periods. The center of each square represents the weighted mean differences for individual trials, and the corresponding horizontal line stands for a 95% confidence interval. The diamonds represent pooled results. Legend: CI = confidence interval; MD = mean difference.

Overall, the time to EMS arrival on the scene, reported in 18 studies, was 9.1 ± 2.1 min in the pre-COVID-19 period versus 9.8 ± 2.6 min (MD = −1.05; 95%CI: −1.54 to −0.56; *p* < 0.001) since the COVID-19 pandemic began (Figure 2).

**Figure 3:**
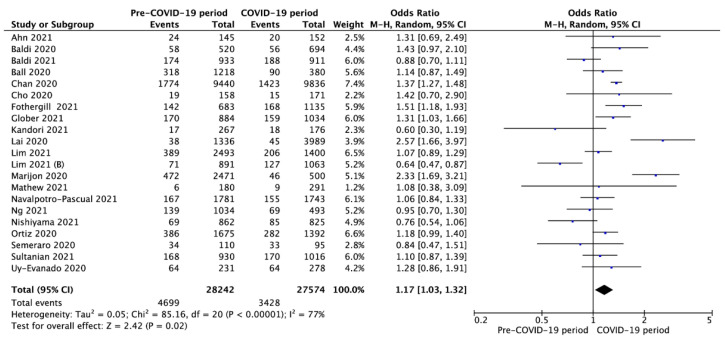
Forest plot of occurrence of shockable rhythm in pre- vs. intra-COVID-19 periods. The center of each square represents the weighted odds ratios for individual trials, and the corresponding horizontal line stands for a 95% confidence interval. The diamonds represent pooled results. Legend: CI = confidence interval; OR = odds ratio.

Shockable initial rhythms were observed in 16.7% of OHCA in the pre-COVID-19 period compared to 12.4% since the COVID-19 pandemic began (OR = 1.17; 95%CI: 1.03 to 1.32; *p* < 0.001; Figure 3).

Pooled analysis of resuscitation outcomes are presented in Table 1.

There was no significant difference between pre-COVID-19 and COVID-19 periods in bystander-witnessed arrests, bystander CPR, use of mechanical chest compression or use of targeted temperature management (*p* > 0.05). However, since the COVID-19 pandemic began, endotracheal intubation was utilized more frequently during resuscitation (OR = 1.91; 95%CI: 1.37 to 2.68; *p* < 0.001), cardiac arrest was observed more frequent at home (OR = 0.74; 95%CI: 0.65 to 0.84; *p* < 0.001) and bystanders used AED less frequently (OR = 1.35; 95%CI: 1.25 to 1.46; *p* < 0.001). A visual examination of the funnel plots did not reveal relevant asymmetry consistent with publication bias (Figures S3 and S4).

### 3.3. Outcomes in Pre- vs. Intra-COVID-19 Periods

**Figure 4:**
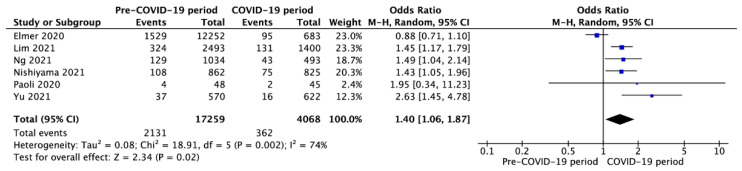
Forest plot prehospital return of spontaneous circulation in pre- vs. intra-COVID-19 periods. The center of each square represents the odds ratios for individual trials, and the corresponding horizontal line stands for a 95% confidence interval. The diamonds represent pooled results. Legend: CI = confidence interval; OR = odds ratio.

Six studies [31,38,44,48,53] reported prehospital ROSC in pre-COVID-19 and COVID-19 periods with a variance of 12.3% vs. 8.9% (OR = 1.40; 95%CI: 1.06 to 1.87; *p* < 0.001; Figure 4), respectively.

**Figure 5:**
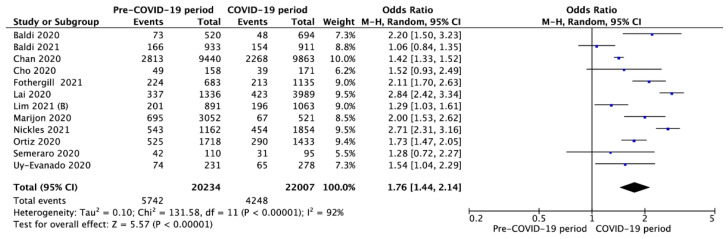
Forest plot of survival to hospital admission rate in pre- vs. intra-COVID-19 periods. The center of each square represents the weighted odds ratios for individual trials, and the corresponding horizontal line stands for a 95% confidence interval. The diamonds represent pooled results. Legend: CI = confidence interval; OR = odds ratio.

Survival to hospital admission with spontaneous circulation was reported in 12 studies and was significantly higher in the pre-COVID-19 period compared to the COVID-19 period (28.4% vs. 19.3%, respectively; OR = 1.76; 95%CI: 1.44 to 2.14; *p* < 0.001; Figure 5).

**Figure 6:**
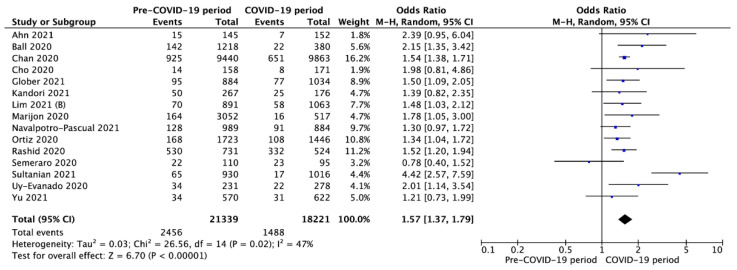
Forest plot of survival to hospital discharge in pre- vs. intra-COVID-19 periods. The center of each square represents the weighted odds ratios for individual trials, and the corresponding horizontal line stands for a 95% confidence interval. The diamonds represent pooled results. Legend: CI = confidence interval; OD = odds ratio.

Fifteen studies reported survival to hospital discharge. Pooled analysis of SHD was 11.5% in OHCA patients in the pre-COVID-19 period compared to 8.2% during the COVID-19 period (OR = 1.57; 95%CI: 1.37 to 1.79; *p* < 0.001; Figure 6). A funnel plot and Egger’s regression test were employed to examine publication bias (Figure S5).

**Figure 7:**
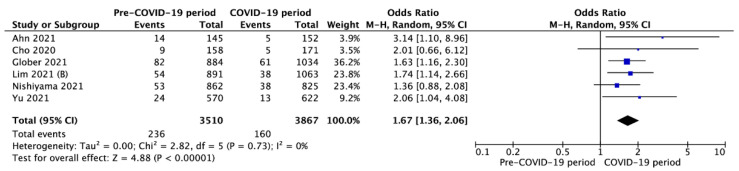
Forest plot of survival to hospital discharge with CPC 1 or 2 in pre- vs. intra-COVID-19 periods. The center of each square represents the weighted odds ratios for individual trials, and the corresponding horizontal line stands for a 95% confidence interval. The diamonds represent pooled results. Legend: CI = confidence interval; OD = odds ratio.

**Figure 8:**
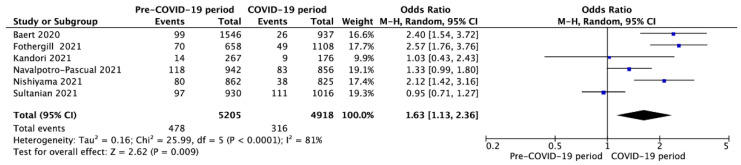
Forest plot of 30-day survival rate in pre- vs. intra-COVID-19 periods. The center of each square represents the weighted odds ratios for individual trials, and the corresponding horizontal line stands for a 95% confidence interval. The diamonds represent pooled results. Legend: CI = confidence interval; OR = odds ratio.

Survival to hospital discharge with CPC 1-2 was reported in six studies and was 6.7% vs. 4.0% for pre-COVID-19 and during the COVID-19 periods (OR = 1.71; 95%CI: 1.35 to 2.15; *p* < 0.001; Figure 7). Moreover, six studies reported a 30-day survival rate in pre-COVID-19 and COVID-19 periods with a variance of 9.2% vs. 6.4%, respectively (OR = 1.63; 95%CI: 1.13 to 2.36; *p* = 0.009; Figure 8). A visual examination of the funnel plots did not reveal relevant asymmetry consistent with publication bias (Figure S6).

### 3.4. Outcomes in SARS-CoV-2-Positive vs. -Negative Patients

Seven studies showed survival outcomes in during the pandemic among patients with and without SARS-CoV-2 infection [25,30,32,34,36,43,51]. Pooled analysis of ROSC in SARS-CoV-2 positive vs. negative patients was 22.9% vs. 28.3%, respectively (OR = 0.69; 95%CI: 0.52 to 0.92; *p* = 0.01; Table 2). A similar relationship was observed in both survival to hospital admission (8.8% vs. 18.5%; OR = 0.44; 95%CI: 0.22 to 0.88; *p* = 0.02), as well as in the 30-day survival ratio (0.7% vs. 4.2%; OR = 0.12; 95%CI: 0.05 to 0.31; *p* < 0.001). There were no significant differences between SARS-CoV-2-positive and -negative patients in the context of survival to hospital discharge (1.7% vs. 4.2%; OR = 0.98; 95%CI: 0.25 to 3.83; *p* = 0.97), as well as in survival to hospital discharge with CPC 1 or 2 (11.1% vs. 3.8%; OR = 2.67; 95%CI: 0.47 to 15.28; *p* = 0.27).

## 4. Discussion

The time of arrival of EMS teams on site was longer during the COVID-19 period. This parameter is a critical predictor of resuscitation success, as every minute of delay significantly reduces the survival chances [48,54]. Our findings are consistent with those reported by Yu [53], who showed that not only were response times longer since COVID-19 began but that the rapid deployment of advanced lifesaving procedures was crucial to patient survival. This was noted in Italy and Spain as well, where EMS response time was prolonged. However, in these environments, the authors emphasize that an increased volume of calls may have been the cause of these delays [47], as was the case in Detroit [45]. Because the initial response is vital to increasing the chance of a good outcome in OHCA patients [55], a public health initiative to encourage people to perform CPR [56] may improve survival [24]. Interestingly, a study from Osaka Japan found that although the bystander CPR, as well as AED usage, was lower during the COVID period, the outcomes measured by the 1-month survival with favorable neurological outcomes did not change [46].

We found a significant difference in shockable rhythms between pre-COVID-19 and COVID-19 patients; shockable rhythm is less frequent during the COVID-19 pandemic. This finding is worrisome, as the shockable rhythms have a more favorable prognosis [57]. Because of the risk of contracting SARS-CoV-2, some authors have suggested that, in patients >60 years old with nonshockable rhythms, resuscitation should be terminated early due to the high risk of contamination of the EMS team [58]. Additionally, Baldi et al. reported the decrease in bystander CPR and subsequent decline in EMS provided resuscitation over the analyzed periods of 2019 and 2020 [26].

Interestingly we found that the use of advanced airway management techniques was more prevalent since COVID-19, both in the form of endotracheal intubation, as well as supraglottic airway devices. This finding is particularly interesting, as initial guidelines placed high value on the avoidance of airway manipulation to avoid aerosol formation [59], which increases the risk of SARS-CoV-2 transmission [60]. However, more recent data show that although intubation generates aerosols, it poses a low risk of infection when performed by an experienced healthcare provider following proper protocols [61,62]. Additionally, simulation studies show that intubation is a safe procedure, provided that the healthcare professional is wearing PPE. However, to complicate the analysis, the wearing of PPE during the procedure may itself result in prolonged time to resuscitation [63,64,65].

When analyzing time to ROSC, we found that it was longer before COVID-19. Semeraro [50] confirmed this finding in his data analysis as well. The reason behind these results may lie in the coexisting respiratory arrest [66]. Other studies also report lowering of the time to ROSC since COVID-19 [29,44] due to this phenomenon. Interestingly when analyzing the Detroit population, Shinobi found no difference in ROSC between COVID and pre-COVID-19 periods [41].

In the analyzed population, the survival to hospital admission with spontaneous circulation was significantly higher before COVID-19. However, we must note that over the course of COVID-19, many patients did not receive advanced life support (ALS), therefore further reducing their survival chances [67,68]. The analysis by Sultanian et al. [51] showed more than a threefold increase in 30-day mortality among COVID-19-positive patients. Additionally, during the initial phase of the COVID-19 pandemic, many patients may have been afraid to seek help despite coronary symptoms, which could have prolonged the treatment and diagnostic process, thus resulting in worse outcomes [69].

Both survival to overall hospital discharge and survival to hospital discharge with CPC 1-2 were higher in the pre-pandemic period. The neurological damage resulting from cardiac arrest [70] is further exacerbated by the COVID-19 infection [71]. These findings are in line with those achieved by Glober et al. [33], who also underlined that patients were more likely to die in the field, with those who survive having a worse neurological status. The data provided by Ahn further reinforce these conclusions [23]. In the patient group analyzed by Cho, not a single patient who was diagnosed with COVID achieved a favorable neurological outcome [30].

## 5. Conclusions

In the COVID-19 era, the prognosis of OHCA is worse. Moreover, cardiac arrests occurred more frequently at home, and bystanders used AED less frequently.

## Figures and Tables

**Table 1:** Resuscitation characteristics in pre- vs. intra-COVID-19 periods.

Outcome	No. of Studies	Events/Participants		Events		Heterogeneity between Trials		*p*-Value for Differences across Groups
		Pre-COVID-19 Period	COVID-19 Period	OR	95%CI	*p*-Value	I^2^ Statistic	
Cardiac arrest location at home	17	19,493/26,948 (73.3%)	19,860/25,625 (77.5%)	0.74	0.65 to 0.84	<0.001	86%	<0.001
Witnessed arrest	21	16,798/37,960 (44.3%)	12,416/26,994 (46.0%)	1.04	0.96 to 1.11	<0.001	62%	0.34
Bystander CPR	23	17,092/38,741 (44.1%)	12,586/27,248 (46.2%)	1.00	0.88 to 1.14	<0.001	90%	1.0
Bystander AED use	14	1704/21,089 (8.1%)	1221/19,964 (6.1%)	1.35	1.25 to 1.46	<0.001	73%	<0.001
Advanced airway management	10	9707/20,839 (46.6%)	8166/12,549 (65.1%)	1.20	0.82 to 1.76	<0.001	97%	0.34
Endotracheal intubation	8	6605/20,058 (32.9%)	3838/10,277 (37.3%)	1.91	1.37 to 2.68	<0.001	95%	<0.001
Supraglottic airway devices	8	2926/19,410 (15.1%)	3743/10,519 (35.6%)	0.67	0.42 to 1.05	<0.001	97%	0.08
Mechanical chest compression	3	486/2629 (18.5%)	557/2137 (26.1%)	0.97	0.50 to 1.88	<0.001	92%	0.93
Targeted temperature management	3	81/2920 (2.8%)	44/2638 (1.7%)	1.62	0.85 to 3.07	0.07	63%	0.14

Legend: AED: automated external defibrillation; CI: confidence interval; CPR: cardiopulmonary resuscitation; OR: odds ratio. Note: Not all outcomes were reported in every study. “No. of studies” refers to the studies included in the analysis for the particular outcome.

**Table 2:** Survival outcomes among SARS-CoV-2-positive vs. -negative patients.

Outcome	No of Studies	Events/Participants		Events		Heterogeneity between Trials		*p*-Value for Differences across Groups
		SARS-CoV-2 (+)	SARS-CoV-2 (−)	OR	95%CI	*p*-Value	I^2^ Statistic	
ROSC	6	173/757 (22.9%)	582/2058 (28.3%)	0.69	0.52 to 0.92	0.23	27%	0.01
SHA	4	51/582 (8.8%)	277/1498 (18.5%)	0.44	0.22 to 0.88	0.07	58%	0.02
SHD	4	2/115 (1.7%)	25/591 (4.2%)	0.98	0.25 to 3.83	0.37	5%	0.97
SHD with CPC 1-2	2	2/18 (11.1%)	7/186 (3.8%)	2.67	0.47 to 15.28	0.58	0%	0.27
30-day survival	3	4/606 (0.7%)	299/7055 (4.2%)	0.12	0.05 to 0.31	0.63	0%	<0.001

Legend: CI = confidence interval; CPC = Cerebral Performance Categories Scale; OR = odds ratio; ROSC = return of spontaneous circulation; SHA = survival to hospital admission; SHD = survival to hospital discharge. Note: Not all outcomes were reported in every study. “No. of studies” refers to the studies included in the analysis for the particular outcome.

## Data Availability

Not applicable.

## Supplementary Materials

The following are available online at https://www.mdpi.com/article/10.3390/jcm10235573/s1, Table S1: Characteristics of included studies, Table S2: Methodology characteristics of included trials, Table S3: PRISMA checklist, Figure S1: A summary table of review authors’ judgments for each risk of bias item for each study, Figure S2: A plot of the distribution of review authors’ judgments across studies for each risk of bias item, Figure S3: Funnel plot to illustrate possible publication bias due to time to Emergency Medical Service arrival, Figure S4: Funnel plot to illustrate possible publication bias due to occurrence of shockable rhythm, Figure S5: Funnel plot to illustrate possible publication bias due to survival to hospital admission, Figure S6: Funnel plot to illustrate possible publication bias due to survival to hospital discharge.
