# Supplementary material for: The Influence of COVID-19 on Out-Hospital Cardiac Arrest Survival Outcomes: An Updated Systematic Review and Meta-Analysis
Source: J Clin Med. 2021 Nov 27;10(23):5573. doi: 10.3390/jcm10235573 (PMC8658174; doi:10.3390/jcm10235573)
Supplement: Supplementary file 1 [file jcm-10-05573-s001.zip › jcm-1461634-supplementary.pdf]

The Influence of COVID-19 on Out-Hospital Cardiac Arrest Survival Outcomes: An Updated Systematic Review and Meta-Analysis

Supplementary Digital File

Content:

Table S1. Characteristics of included studies..2

Table S2. Methodology characteristics of included trials. 5

Table S3. PRISMA checklist..12

Figure S1. A summary table of review authors' judgements for each risk of bias item for each study...15

Figure S2. A plot of the distribution of review authors' judgements across studies for each risk of bias item. 16

Figure S3. Funnel plot to illustrate possible publication bias due to time to Emergency Medical Service arrival. 16

Figure S4. Funnel plot to illustrate possible publication bias due to occurrence of shockable rhythm...17

Figure S5. Funnel plot to illustrate possible publication bias due to survival to hospital admission...17

Figure S6. Funnel plot to illustrate possible publication bias due to survival to hospital discharge. 18

Table S1. Characteristics of included studies.

Study	Country	Pre-COVID-19 Period			COVID-19 Period								
					Total			COVID-19 Positive Patients			COVID-19 Negative Patients		
		No.	Age	Sex, Male	No.	Age	Sex, Male	No.	Age	Sex, Male	No.	Age	Sex, Male
**Ahn et al. 2021**	Korea	145	72.9 ±3.4	91 (62.8%)	152	75 ±2.6	102 (67.1%)	–	–	–	–	–	–
Baert et al. 2020	France	1,620	69 ±17	1,071 (66.1%)	1,005	68 ±17	676 (67.3%)	–	–	–	–	–	–
Baert et al. 2021	France	NR	NR	NR	6,151	67.9 (4.2)	4,168 (67.8%)	127	71± 4	76 (59.8%)	6,024	67.8± 4.2	4,092 (67.9%)
Baldi et al. 2020	Italy	520	77.3 ±3.5	300 (57.7%)	694	76.5 ±3	430 (62.0%)	–	–	–	–	–	–
Baldi et al. 2021	Switzerland	933	70.5 ±4	636 (68.2%)	911	69 ±4	623 (68.4%)	–	–	–	–	–	–
Ball et al. 2020	Australia	1,218	66 ±4.3	845 (69.4%)	380	68 ±4.3	250 (65.8%)	–	–	–	–	–	–
Chan et al. 2020	USA	9,440	62.2 ±19.2	5,922 (62.7%)	9,863	62.6 ±19.3	6,040 (61.3%)	–	–	–	–	–	–
Cho et al. 2020	Korea	158	73.2 ±3.4	103 (65.2%)	171	72.5 ±3	121 (70.8%)	10	73.3 (4.3)	4 (40.0%)	161	72.3 (5.5)	104 (64.6%)
Elmer et al. 2020	USA	12,252	63 ±19	7,700 (62.8%)	683	64 ±19	430 (63.0%)	–	–	–	–	–	–
Fothergill et al. 2021	United Kingdom	1,724	68 ±20	1,069 (62.0%)	3,122	71 ±19	1,839 (59.0%)	766	70 (18)	468 (61.2%)	2,356	71 (19)	1,371 (58.3%)
Glober et al. 2021	USA	884	64 ±2.3	554 (62.7%)	1,034	59.8 ±4.2	622 (60.2%)	–	–	–	–	–	–
Hubert et al. 2020	France	NR	NR	NR	670	68.2 (17.2)	461 (68.8%)	146	67.5 (17.5)	88 (60.3%)	524	68.4 (17.1)	373 (71.2%)

Kandori et al. 2021	Japan	267	76±3.3	164 (61.4%)	176	75±3.3	97 (55.1%)	–	–	–	–	–	–
Kim et al. 2020	Korea	NR	NR	NR	80	74.1 ±2.7	46 (57.5%)	9	74.1± 4.7	3 (33.3%)	71	74± 4.6	43 (60.6%)
Lai et al. 2020	USA	1,336	68 ±19	752 (57.1%)	3,989	72 ±18	2,183 (55.8%)	–	–	–	–	–	–
Lim et al. 2021	Singapore	2,493	71 ±3.8	1,597 (64.1%)	1,400	72.5 ±4	882 (63.0%)	–	–	–	–	–	–
Lim et al. 2021 (B)	Korea	891	70.07 (15.06)	577 (64.8%)	1,063	71.05 (14.98)	647 (60.9%)	–	–	–	–	–	–
Marijon et al. 2020	France	3,047	68.5 ±18	1,826 (59.9%)	519	69.7 ±17	334 (64.4%)	–	–	–	–	–	–
Mathew et al. 2021	USA	180	58.5 ±19.8	93 (51.7%)	291	64.5 ±18.1	165 (56.7%)	–	–	–	–	–	–
Navalpotro -Pascual et al. 2021	Spain	1,781	71.3 (3.8)	1,178 (66.1%)	1,743	70 (4)	1,117 (64.0%)	–	–	–	–	–	–
Navalpotro -Pascual et al. 2021 (B)	Spain	–	–	–	313	71.8 (3.3)	189	87	70 (2.7)	54	226	72.5 (3.3)	135
Ng et al. 2021	Singapore	1,034	73.2 ±4	NS	493	72.4 ±4	NS	–	–	–	–	–	–
Nickles et al. 2021	USA	1,162	NS	662 (57.0%)	1,854	NS	1,083 (58.4%)	–	–	–	–	–	–
Nishiyama et al. 2021	Japan	862	74 ±3.3	551 (63.9%)	825	76.3 ±3.2	529 (64.1%)	–	–	–	–	–	–
Ortiz et al. 2020	Spain	1,723	65.61 ±16.9	1,208 (70.1%)	1,446	64.36 ±16.5	1,027 (71.0%)	–	–	–	–	–	–
Paoli et al. 2020	Italy	206	77 ±14	98 (54.7%)	200	79 ±17	89 (50.9%)	–	–	–	–	–	–
Rashid et al. 2020	United Kingdom	731	63.1 ±12.2	581 (79.5%)	524	67.1 ±13.2	373 (71.2%)	–	–	–	–	–	–

Semeraro et al. 2020	Italy	563	83 ±3	284 (50.4%)	624	83 ±3	318 (51.0%)	–	–	–	–	–	–
Sultanian et al. 2021	Sweden	930	70.8 ±16.6	604 (64.9%)	1,016	69.6 ±17.8	697 (68.6%)	88	66.5 (18.4)	59	334	70.6 (16.4)	305
Uy-Evanado et al. 2021	USA	231	69.1 ±17.4	137 (59.3%)	278	69.4 ±18.3	174 (62.6%)	–	–	–	–	–	–
Yu et al. 2021	Taiwan	570	70.93 ±16.45	353 (61.9%)	622	70.41 ±16.21	394 (63.3%)	–	–	–	–	–	–

Table S2. Methodology characteristics of included trials.

Study	Inclusion Criteria	Exclusion Criteria	Primary Outcome(s)	Findings
Ahn et al. 2021	All adults (aged 18 years or older) presenting with OHCA, with presumed cardiac etiology.	Patients who did not receive resuscitation attempts by EMS, arrests that were witnessed by EMTs, and patients who did not receive resuscitation attempts from the in-hospital medical team owing to a dead on arrival status after arriving at the ED.	Good neurologic outcome, while the secondary outcome was survival to hospital discharge.	During the COVID-19 pandemic, the response and on-scene times were longer, and good neurologic outcome was significantly lower than that in the control period.
Baert et al. 2020	All medical OHCA according to the Utstein template.	Physical indication of death, patients with a known Do Not Attempt Resuscitation (DNAR) order, end of life patients, and traumatic drowning, overdose, asphyxia (external causes) and electrocution OHCA.	Resuscitation characteristic.	During the COVID-19 period, we observed a decrease in CPR initiation regardless of whether patients were suspected of SARS-CoV-2 infection or not. In the current atmosphere, it is important to communicate good resuscitation practices to avoid drastic and lasting reductions in survival rates after an OHCA.
Baert et al. 2021	Cases of OHCA recorded in the RéAC between March 1, 2020, and December 31, 2020.	patients with pro- longed downtime and unwitnessed arrest with signs of rigor mortis and those whose COVID-19 status was not known	The survival rate 30 days after OHCA among confirmed COVID-19 patients.	Our results highlighted a zero survival rate in out-of-hospital cardiac arrest patients with confirmed coronavirus disease 2019. This finding raises important questions with regard to the futility of resuscitation for corona- virus disease 2019 patients and the management of the associated risks.
Baldi et al. 2020	All OHCA cases.	NS	OHCA characteristics	Compared to 2019, during the 2020 COVID-19 outbreak we observed a lower attitude of lay-people to start CPR, while resuscitation attempts by BLS and ALS staff were not influenced by suspected/confirmed infection, even at univariable analysis.
Baldi et al. 2021	All OHCA cases.	EMS witnessed OHCA events and patients for whom a Do-Not-Resuscitate	Prehospital ROSC	During the COVID-19 pandemic in Switzerland mortality increased in Cantons with high-incidence of infection, whilst not in the low-

		order/Advanced Care Directive was in place.		incidence ones. OHCA occurrence followed an opposite trend showing how variables related to the health-system and EMS organization deeply influence OHCA occurrence during a pandemic.
Ball et al. 2020	Adult OHCA patients (aged>15 years) attended by paramedics.	EMS witnessed OHCA events and patients for whom a Do-Not-Resuscitate order/Advanced Care Directive was in place.	Arrest incidence, characteristics and survival rates.	The COVID-19 pandemic period did not influence OHCA incidence but appears to have disrupted the system-of-care in Australia. However, this could not completely explain reductions in survival.
Chan et al. 2020	Adults patients with OHCA.	OHCAs witnessed by EMS.	Sustained ROSC (=>20 minutes), survival to discharge, and OHCA incidence.	Early during the pandemic, rates of sustained ROSC for OHCA were lower throughout the US, even in communities with low COVID-19 mortality rates. Overall survival was lower, primarily in communities with moderate or high COVID-19 mortality.
Cho et al. 2020	Patients who were aged 18 years or older with OHCA of presumed medical aetiology and who used the EMS system in Daegu.	Patients who did not undergo resuscitative attempts and cases in which cardiac arrest occurred in a primary care clinic or long-term care hospital.	Demographic and clinical characteristics, overall survival, COVID-19 related data.	Responses to the COVID-19 pandemic included changes to current PPE strategies and introduction of isolated resuscitation units; the latter intervention reduced the number of unexpected closures and quarantines of emergency resources early on during the COVID-19 outbreak. Given the possibility of future outbreaks, we need to have revised resuscitation strategies and the capacity to commandeer emergency resources for OHCA patients.
Elmer et al. 2020	Adults >18 years assessed by one of 24 regional EMS agencies for OHCA.	Interfacility transfers and duplicate charts generated by multiple responding units.	OHCA characteristics.	Multiple small but significant differences in patient care, including airway management and time to epinephrine administration, are of unclear significance but merit further systematic investigation.
Fothergill et al. 2021	All OHCA patients who, during the study periods, received an EMS response from LAS (irrespective of whether a	Patients successfully resuscitated prior to EMS arrival and so did not receive resuscitation from our clinicians.	ROSC at any point, ROSC sustained to hospital, and survival to 30 days post-event.	During the first wave of the COVID-19 pandemic in London, we saw a dramatic rise in the incidence of OHCA, accompanied by a significant reduction in survival. The pattern of increased incidence and mortality closely

	resuscitation attempt was made).			reflected the rise in confirmed COVID-19 infections in the city.
Glober et al. 2021	All OHCA is routinely collected for quality assurance by EMS from the electronic medical record.	All patients with incomplete data.	Variability in the volume, characteristics, and management of OHCA during the COVID-19 pandemic in a major EMS system compared to the previous year.	Total OHCA increased during the COVID-19 pandemic when compared with the prior year. Although patient characteristics were similar, initial shockable rhythm, and proportion of patients who died in the hospital decreased during the pandemic.
Hubert et al. 2020	This retrospective multicentre cohort study, we collected OHCA data, from 20 MERSs (Argenteuil, Aulnaysous-Bois, Bobigny, Corbeil-Essonnes, Creteil, Douai, Garches, Grenoble, Lyon, Melun, Montfermeil, Nantes, Orléans, Rennes, Roanne, Roubaix, Selestat, Saint-Denis, Tourcoing, and Troyes) in towns and cities throughout France.	NS	The incidence and survival at hospital admission of cases of COVID-19 OHCA occurring at home.	The ratio of COVID-19 out-of-hospital deaths to in-hospital deaths was 12.4%, and so the national statistics underestimated the death rate.
Kandori et al. 2021	Adult patients (age ≥ 18 years) with OHCA who arrived at our emergency department under CPR between January 1, 2019, and December 31, 2020.	OHCA patients who had a return of spontaneous circulation (ROSC) at hospital arrival.	Survival of hospitalization, which was defined as survival at the admission to intensive care or high care unit after the resuscitation and initial evaluation and treatment.	There is no significant change in hospitalization survival outcomes between OHCA patients treated by conventional CPR and those treated under the current measures for controlling COVID-19 transmission.
Kim et al. 2020	Cases of emergency medical service (EMS)–treated adult OHCA.	Vital signs were absent on arrival or there was data duplication (i.e. revisit).	General patient characteristics and hematological findings of the COVID-19-negative and COVID-19-positive groups.	The screening tools that combined fever or abnormal CXR had a good discriminatory ability for COVID-19 infection in adult patients with OHCA. Therefore, during the COVID-19 outbreak period, it is recommended to suspect COVID-19 infection and perform COVID-19 test

				if patients present with a history of fever or show abnormal findings in postmortem CXR.
Lai et al. 2020	Patients 18 years or older with out-of-hospital cardiac arrest who received EMS resuscitation.	Patients with out-of-hospital cardiac arrests were excluded if they did not undergo prehospital CPR owing to obvious signs of death or had a valid do-not resuscitate order present at the time of arrest.	Characteristics associated with out-of-hospital arrests and the outcomes of out-of-hospital cardiac arrests.	In this population-based, cross-sectional study, out-of-hospital cardiac arrests and deaths during the COVID-19 pandemic significantly increased compared with the same period the previous year and were associated with older age, nonwhite race/ethnicity, hypertension, diabetes, physical limitations, and nonshockable presenting rhythms. Identifying patients with the greatest risk for out-of-hospital cardiac arrest and death during the COVID-19 pandemic should allow for early, targeted interventions in the outpatient setting that could lead to reductions in out-of-hospital deaths.
Lim et al. 2021	adult (18 years or older), EMS-attended OHCA of all etiologies occurring in Singapore between 1 January and 31 May in the years of 2018, 2019 and 2020.	NS	Pre-hospital ROSC	The pandemic saw increased OHCA incidence and worse outcomes in Singapore, likely indirect effects of COVID-19.
Lim et al. 2021 (B)	All patients with OHCA.	Patients with less than 18 years old, if resuscitation was not attempted due to obvious signs of death or if they had a valid do-not-resuscitate order. Patients who were pulseless because of trauma, intoxication, or drowning.	The neurological outcome of OHCA patients.	During the COVID-19 period, there were significant changes in the EMS responses to OHCA. These changes are considered to be partly due to social distancing measures. As a result, the proportion of patients with an initial shockable rhythm in the COVID-19 period was greater than that in the pre-COVID-19 period, but the final survival rate and favorable neurological outcome were lower.
Marijon et al. 2020	All cases of sudden OHCA occurring among adults (aged 18 years and older) in the city of Paris and its three suburbs.	Cases with obvious accidental causes, irrespective of whether resuscitation was attempted or no.	Patient characteristics, setting of occurrence.	A transient two-times increase in OHCA incidence, coupled with a reduction in survival, was observed during the specified time period of the pandemic when compared with the equivalent time period in previous years with no pandemic. Although this result might be partly related to COVID-19 infections, indirect effects associated with

				lockdown and adjustment of health-care services to the pandemic are probable. Therefore, these factors should be taken into account when considering mortality data and public health strategies.
Mathew et al. 2021	Patients 18 years and older with non-traumatic OHCA .	patients if no resuscitation was attempted or the arrest was deemed to be traumatic in natur	ROSC, patient demographics, arrest location, initial rhythms, bystander CPR and field termination were compared before and during the pandemic	OHCA increased by 62% during COVID-19 in Detroit,without a significant change in prehospital ROSC.<br>The rate of ROSC remained similar despite the implementation of an early termination of resuscitation protocol in response to COVID-19.
Navalpotro-Pascual et al. 2021	All patients with OHCA.	NS	Hospital admission with return of spontaneous circulation, overall survival to hospital discharge, and overall survival with a good neurological outcome	The different phases of the pandemic variably affected OHCA care. The first wave led to longer resource activation,<br>increased home events and scene arrival times, as well as lower patient survival.
Navalpotro-Pascual et al. 2021 (B)	All patients with OHCA.	NS	The time of activation and arrival to the place from the call; the variable COVID; whether the PCR was witnessed or not; if previous CPR was performed by the first respondent and if advanced CPR was performed, as well as the reason why advanced CPR was not performed; if there was advanced CPR, the first	Mortality was higher in cases of cardiopulmonary arrest during the COVID-19 pandemic. The percentage of cases with no application of advanced CPR rose; the main reason was the amount of time between collapse and first response. Even though the number of emergency calls increased significantly, the SUMMA112 service did not dispatch more ambulances.

			rhythm detected, if the airway was isolated and method used, if the patient arrived alive at the hospital and his / her survival at discharge and at one month.	
Ng et al. 2021	OHCA data collected by the Singapore Civil Defence Force on EMS utilisation in Singapore from 1 April to 31 May 2020.	NS	Prehospital ROSC.	The findings contribute to a growing body of literature internationally on the potential impact of the COVID-19 pandemic on EMS utilisation and outcomes.
Nickles et al. 2021	Nontraumatic OHCA calls in Macomb, Oakland, and Wayne counties between January 1 and May 31 of both 2019 and 2020 were identified from the Michigan EMS Information System.	NS	Clinical characteristics of OHCA events.	Southeast Michigan experienced marked increases in both the number of OHCA calls and the prehospital fatality rates for OHCA. In 2020, OHCA disproportionately increased among older individuals, Blacks, and residents of skilled nursing facilities.
Nishiyama et al. 2021	OHCA cases treated by EMS personnel according to the Utstein-style guidelines in Osaka City.	No resuscitation attempt, traumatic cases, cases that occurred in health care facilities (e.g., nursing home and long-term care facilities), or cases witnessed by EMS personnel were excluded. Since EMS personnel were not allowed to terminate resuscitation in a prehospital setting, all OHCA cases were transported to a medical institution and registered in registry.	Survival outcomes.	This population-based study showed that the COVID-19 pandemic did not affect outcomes after OHCA but appeared to change bystander behaviors, such as CPR and public-access AED pad application.
Ortiz et al. 2020	All consecutive OHCA cases in which an emergency team	Cases were excluded if the emergency team suspended	Overall survival from OHCA to hospital	The pandemic, irrespective of its incidence, seems to have particularly impeded the pre-

	performed resuscitation manoeuvres or postresuscitation care following cardiopulmonary resuscitation (CPR) attempts by a first responder.	resuscitation on-site due to confirmation of futility criteria during resuscitation.	admission and discharge.	hospital phase of OHCA care. Present findings call for the need to adapt out-of-hospital care for periods of serious infection risk.
Paoli et al. 2020	All patients with OHCA.	NS	Survival outcomes.	Compared with findings from Lombardy and Paris, we did not observe an increase in OHCA incidence and mortality, in line with what reported in Seattle and King County, USA. Instead, a delay in EMS arrival and a numerical reduction in bystander-CPR rate was observed.
Rashid et al. 2020	Patients, aged 18 to 100 years, admitted with a diagnosis of AMI between February 1, 2019, and May 14, 2020 (the latest live data upload available), from the MINAP registry and BCIS registry PCI database.	Exclusions were made on the basis of missing record information on sex, cardiac arrest in hospital, and final diagnosis not being AMI.	The characteristics, care, and outcomes of admissions to hospital with AMI complicated by OHCA during the first wave of the COVID-19 outbreak.	In this national cohort of hospitalized patients with acute myocardial infarction, we observed a significant increase in incidence of OHCA during COVID-19 period paralleled with reduced access to guideline-recommended care and increased in-hospital mortality.
Semeraro et al. 2020	All patients with OHCA.	NS	Survival outcomes.	With the limit of this data sample, we could not demonstrate that in our area there was a decrease in terms of EMS's performances in response to OHCA when resuscitation was attempted. It will be essential to analyze the quality of the data in order to measure the "real" _impact of COVID19 on cardiac arrest incidence. This could be another "mission" _for EuReCa network under the umbrella of European Resuscitation Council Research NET.
Sultanian et al. 2021	All cases of OHCA registered in the SRCR from 1 January to 20 July 2020.	Patients enrolled during the pandemic before data on COVID-19.	Overall mortality and 30-day.	During the pandemic phase, COVID-19 was involved in at least 10% of all OHCAs and 16% of IHCAs, and, among COVID-19 cases, 30-day mortality was increased 3.4-fold in OHCA and 2.3-fold in IHCA.

Uy-Evanado et al. 2021	Cases with resuscitation attempted by EMS.	Not meet OHCA criteria.	Survival outcomes.	The community response to OHCA was altered from March to May 2020, with less bystander CPR, delays in EMS response time, and reduced survival from OHCA. These results highlight the pandemic's indirect negative impact on OHCA, even in communities with relatively low incidence of COVID-19 infection, and point to potential opportunities for countering the impact.
Yu et al. 2021	All patients with OHCA.	Patients for whom resuscitation was not attempted, those aged younger than 20 years and those with OHCA as a result of trauma.	The demographical characteristics included on the Utstein-style template were age, sex, OHCA location, presence of witnesses, bystander CPR and AED use status	EMS response time for patients with OHCA was prolonged during the COVID-19 pandemic. Early advanced life support by EMS personnel remains crucial for patients with OHCA.

Legend: CPR = cardiopulmonary resuscitation; DNAR = Do not attempt resuscitation; ED = Emergency Department; EMS = emergency medical service; EMT = emergency medical technician; OHCA = out-of-hospital cardiac arrest; ROSC = return of spontaneous circulation.

Table S3. PRISMA checklist.

Section and Topic	Item #	Checklist Item	Location Where Item Is Reported
		**TITLE**	
Title	1	Identify the report as a systematic review.	1
		**ABSTRACT**	
Abstract	2	See the PRISMA 2020 for Abstracts checklist.	1
		**INTRODUCTION**	
Rationale	3	Describe the rationale for the review in the context of existing knowledge.	2
Objectives	4	Provide an explicit statement of the objective(s) or question(s) the review addresses.	2
		**METHODS**	
Eligibility criteria	5	Specify the inclusion and exclusion criteria for the review and how studies were grouped for the syntheses.	2,3

Information sources	6	Specify all databases, registers, websites, organisations, reference lists and other sources searched or consulted to identify studies. Specify the date when each source was last searched or consulted.	2,3
Search strategy	7	Present the full search strategies for all databases, registers and websites, including any filters and limits used.	2,3
Selection process	8	Specify the methods used to decide whether a study met the inclusion criteria of the review, including how many reviewers screened each record and each report retrieved, whether they worked independently, and if applicable, details of automation tools used in the process.	2,3
Data collection process	9	Specify the methods used to collect data from reports, including how many reviewers collected data from each report, whether they worked independently, any processes for obtaining or confirming data from study investigators, and if applicable, details of automation tools used in the process.	2,3
Data items	10a	List and define all outcomes for which data were sought. Specify whether all results that were compatible with each outcome domain in each study were sought (e.g. for all measures, time points, analyses), and if not, the methods used to decide which results to collect.	3
	10b	List and define all other variables for which data were sought (e.g. participant and intervention characteristics, funding sources). Describe any assumptions made about any missing or unclear information.	3
Study risk of bias assessment	11	Specify the methods used to assess risk of bias in the included studies, including details of the tool(s) used, how many reviewers assessed each study and whether they worked independently, and if applicable, details of automation tools used in the process.	4
Effect measures	12	Specify for each outcome the effect measure(s) (e.g. risk ratio, mean difference) used in the synthesis or presentation of results.	3,4
Synthesis methods	13a	Describe the processes used to decide which studies were eligible for each synthesis (e.g. tabulating the study intervention characteristics and comparing against the planned groups for each synthesis (item #5)).	3
	13b	Describe any methods required to prepare the data for presentation or synthesis, such as handling of missing summary statistics, or data conversions.	3,4
	13c	Describe any methods used to tabulate or visually display results of individual studies and syntheses.	4
	13d	Describe any methods used to synthesize results and provide a rationale for the choice(s). If meta-analysis was performed, describe the model(s), method(s) to identify the presence and extent of statistical heterogeneity, and software package(s) used.	3,4
	13e	Describe any methods used to explore possible causes of heterogeneity among study results (e.g. subgroup analysis, meta-regression).	3,4
	13f	Describe any sensitivity analyses conducted to assess robustness of the synthesized results.	3,4
Reporting bias assessment	14	Describe any methods used to assess risk of bias due to missing results in a synthesis (arising from reporting biases).	3,4
Certainty assessment	15	Describe any methods used to assess certainty (or confidence) in the body of evidence for an outcome.	4
		**RESULTS**	
Study selection	16a	Describe the results of the search and selection process, from the number of records identified in the search to the number of studies included in the review, ideally using a flow diagram.	4
	16b	Cite studies that might appear to meet the inclusion criteria, but which were excluded, and explain why they were excluded.	4

Study characteristics	17	Cite each included study and present its characteristics.	4,5
Risk of bias in studies	18	Present assessments of risk of bias for each included study.	5
Results of individual studies	19	For all outcomes, present, for each study: (a) summary statistics for each group (where appropriate) and (b) an effect estimate and its precision (e.g. confidence/credible interval), ideally using structured tables or plots.	5-8
Results of syntheses	20a	For each synthesis, briefly summarise the characteristics and risk of bias among contributing studies.	5-8
	20b	Present results of all statistical syntheses conducted. If meta-analysis was done, present for each the summary estimate and its precision (e.g. confidence/credible interval) and measures of statistical heterogeneity. If comparing groups, describe the direction of the effect.	5-8
	20c	Present results of all investigations of possible causes of heterogeneity among study results.	5-8
	20d	Present results of all sensitivity analyses conducted to assess the robustness of the synthesized results.	5-8
Reporting biases	21	Present assessments of risk of bias due to missing results (arising from reporting biases) for each synthesis assessed.	5-8
Certainty of evidence	22	Present assessments of certainty (or confidence) in the body of evidence for each outcome assessed.	5-8
		**DISCUSSION**	
Discussion	23a	Provide a general interpretation of the results in the context of other evidence.	8,9
	23b	Discuss any limitations of the evidence included in the review.	9
	23c	Discuss any limitations of the review processes used.	9
	23d	Discuss implications of the results for practice, policy, and future research.	8,9
		**OTHER INFORMATION**	
Registration and protocol	24a	Provide registration information for the review, including register name and registration number, or state that the review was not registered.	2
	24b	Indicate where the review protocol can be accessed, or state that a protocol was not prepared.	2
	24c	Describe and explain any amendments to information provided at registration or in the protocol.	-
Support	25	Describe sources of financial or non-financial support for the review, and the role of the funders or sponsors in the review.	10
Competing interests	26	Declare any competing interests of review authors.	10
Availability of data, code and other materials	27	Report which of the following are publicly available and where they can be found: template data collection forms; data extracted from included studies; data used for all analyses; analytic code; any other materials used in the review.	10

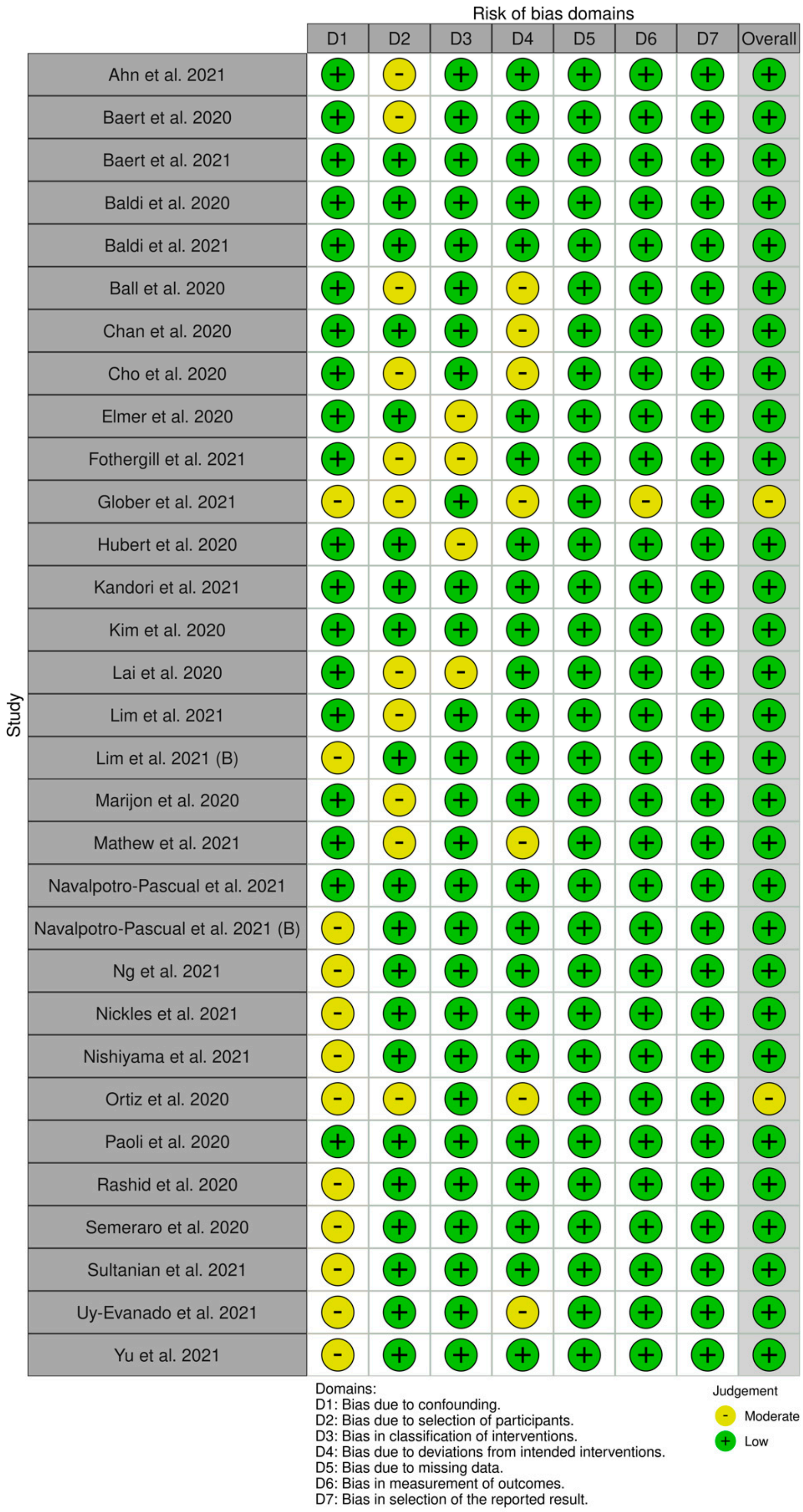

Figure S1. A summary table of review authors' judgements for each risk of bias item for each study.

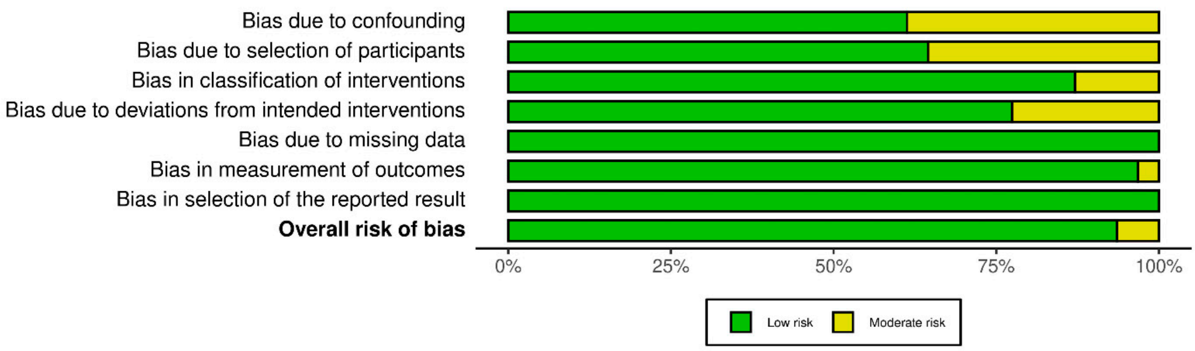

Figure S2. A plot of the distribution of review authors' judgements across studies for each risk of bias item.

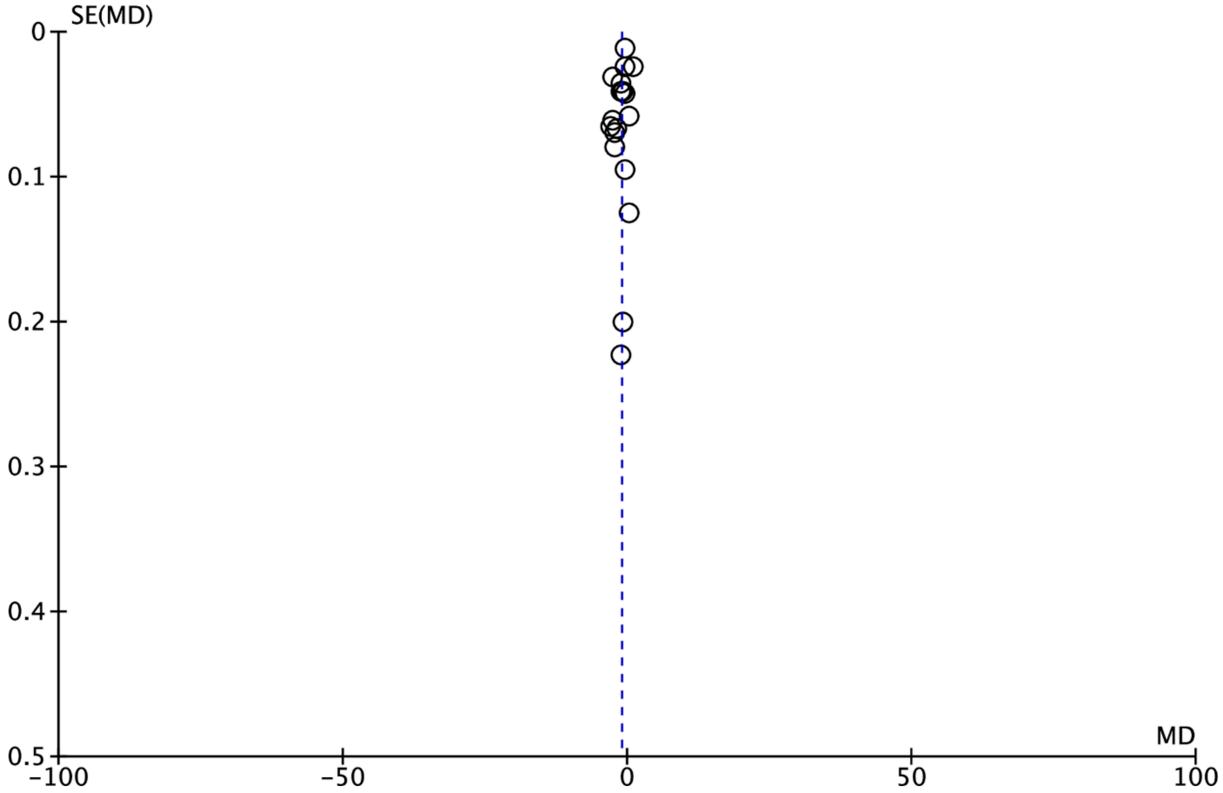

Figure S3. Funnel plot to illustrate possible publication bias due to time to Emergency Medical Service arrival.

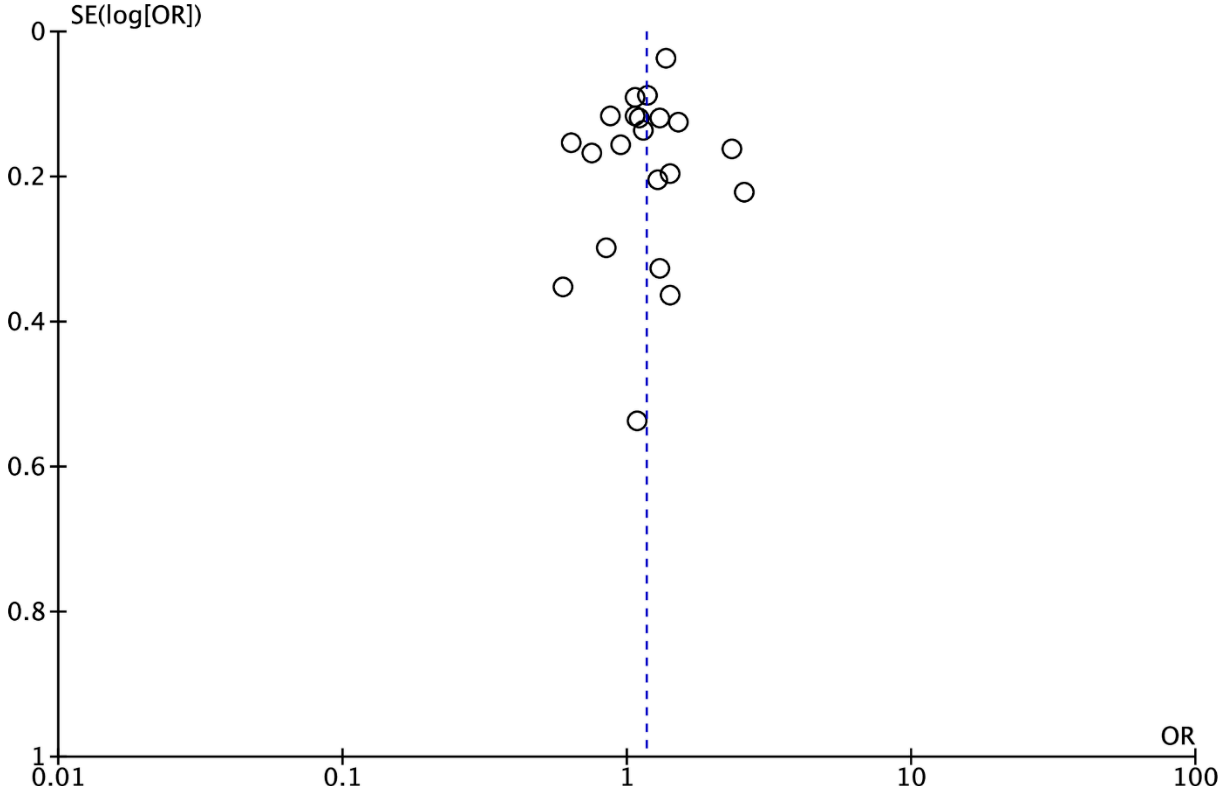

Figure S4. Funnel plot to illustrate possible publication bias due to occurrence of shockable rhythm.

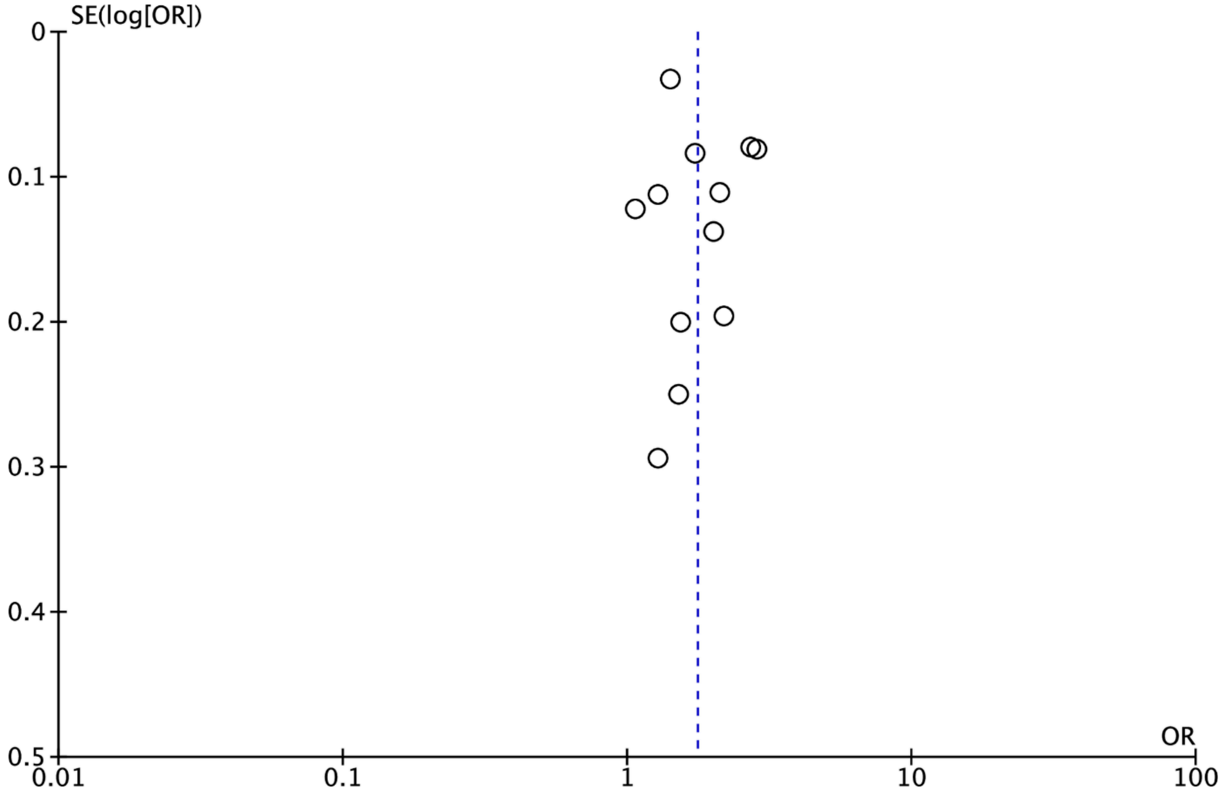

Figure S5. Funnel plot to illustrate possible publication bias due to survival to hospital admission.

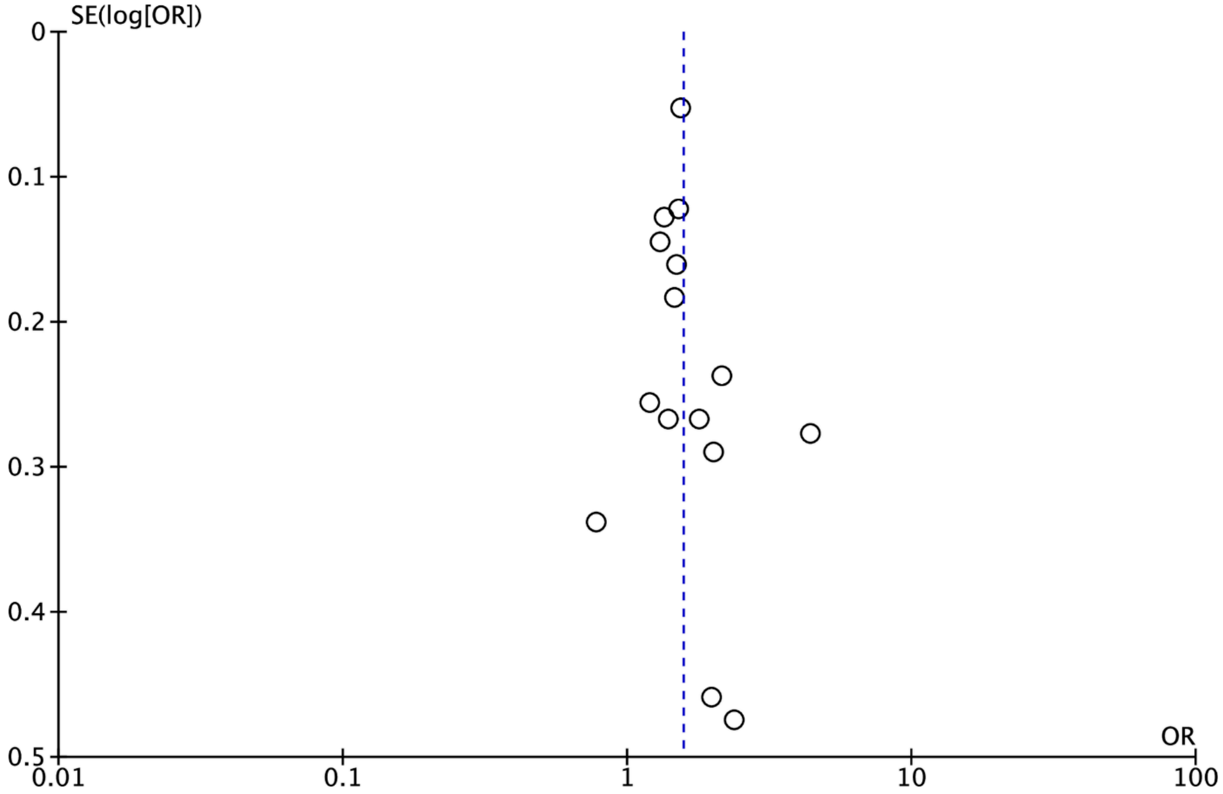

Figure S6. Funnel plot to illustrate possible publication bias due to survival to hospital discharge.
